# The Niche-Derived Glial Cell Line-Derived Neurotrophic Factor (GDNF) Induces Migration of Mouse Spermatogonial Stem/Progenitor Cells

**DOI:** 10.1371/journal.pone.0059431

**Published:** 2013-04-22

**Authors:** Lisa Dovere, Stefania Fera, Margherita Grasso, Dante Lamberti, Cesare Gargioli, Barbara Muciaccia, Anna Maria Lustri, Mario Stefanini, Elena Vicini

**Affiliations:** 1 Fondazione Pasteur Cenci Bolognetti, Department of Anatomical, Histological, Forensic and Orthopaedic Sciences - Section of Histology and Medical Embryology, Sapienza University of Rome, Rome, Italy; 2 Centre for Integrative Biology, University of Trento, Trento, Italy; 3 Department of Biology, University of Rome Tor Vergata, Rome, Italy; University of Bern, Switzerland

## Abstract

In mammals, the biological activity of the stem/progenitor compartment sustains production of mature gametes through spermatogenesis. Spermatogonial stem cells and their progeny belong to the class of undifferentiated spermatogonia, a germ cell population found on the basal membrane of the seminiferous tubules. A large body of evidence has demonstrated that glial cell line-derived neurotrophic factor (GDNF), a Sertoli-derived factor, is essential for in vivo and in vitro stem cell self-renewal. However, the mechanisms underlying this activity are not completely understood. In this study, we show that GDNF induces dose-dependent directional migration of freshly selected undifferentiated spermatogonia, as well as germline stem cells in culture, using a Boyden chamber assay. GDNF-induced migration is dependent on the expression of the GDNF co-receptor GFRA1, as shown by migration assays performed on parental and GFRA1-transduced GC-1 spermatogonial cell lines. We found that the actin regulatory protein vasodilator-stimulated phosphoprotein (VASP) is specifically expressed in undifferentiated spermatogonia. VASP belongs to the ENA/VASP family of proteins implicated in actin-dependent processes, such as fibroblast migration, axon guidance, and cell adhesion. In intact seminiferous tubules and germline stem cell cultures, GDNF treatment up-regulates VASP in a dose-dependent fashion. These data identify a novel role for the niche-derived factor GDNF, and they suggest that GDNF may impinge on the stem/progenitor compartment, affecting the actin cytoskeleton and cell migration.

## Introduction

A paradigm of the adult unipotent stem cell is the spermatogonial stem cell (SSC), which sustains the daily production of millions of mature sperm throughout the male adult life through spermatogenesis. SSCs belong to a class of spermatogonia defined as “undifferentiated” type A spermatogonia, a hallmark of which is their typical nuclear morphology and the expression of markers such as PLZF, neurogenin3, E-cadherin, Lin-28, and GFRA1 [1]; [2]. Spermatogenesis is a cyclic process that in the mouse is divided into 12 stages (I-XII), each stage representing a unique association of germ cells at different steps of differentiation. The relationship between the spermatogenic stages and the kinetics of proliferation and differentiation of the spermatogonia have been analyzed in different mammalian species [2]. In all the stages, undifferentiated spermatogonia can be found as single cells (type Asingle, As) or as interconnected chains of cells composed by two (defined as Apaired: Apr) up to 32 undifferentiated spermatogonia (defined as Aaligned: Aal). Subsequently, during stages VII and VIII of the cycle, almost all of the larger chains (Aal4–Aal32) differentiate into A1 spermatogonia.

In mammals, spermatogonia are located in the basal region of the seminiferous tubules, in contact with the Sertoli cells and basement membrane that separate them from the peritubular myoid cells. Interestingly, spermatogonia are not immotile, they change their relative position. Migration of undifferentiated spermatogonia was first suggested by detailed morphological analysis of the topography of spermatogonia in the mouse testis [3]. More recently, this conclusion was supported by a time-lapse analysis of GFP-labeled undifferentiated spermatogonia that were tracked in vivo for several days and were found to migrate over the basal lamina [4]; [5]. Migration of undifferentiated spermatogonia could ensure even distribution of germ cell progeny over the basal compartment of the seminiferous tubules [3] or may be essential to keeping stem or progenitor cells in the right environment to ensure the self-renewal of the SSCs [6].

Cell migration may be random or directed toward a chemoattractant gradient. Direct migration, or chemotaxis, is activated by extracellular ligands that bind to cell surface receptors, and this process can lead to reorganization of the actin and myosin cytoskeletons and, finally, to cell movement. It has been recently shown that Sertoli cells chemoattract only stem/progenitor spermatogonia and not other germ cell types. In mice with targeted disruption of Ets variant gene 5 (Etv5), the first wave of spermatogenesis is unaffected. With time, the testes of knockout animals show loss of stem/progenitor spermatogonia, resulting in a Sertoli cell-only phenotype. Remarkably, the chemoattractant ability of Sertoli cells isolated from Etv5 knockout animals was decreased compared to wild-type Sertoli cells, suggesting that loss of stem/progenitor cells was due to a decreased chemoattractant ability of Sertoli cells [6].

A large body of evidence has demonstrated that Sertoli-derived GDNF, the ligand for GFRA1, is important for the regulation of self-renewal and differentiation of spermatogonial stem cells, both in vivo and in vitro [7]–[9]. We have recently found that expression and secretion of GDNF is stage-dependent in the mouse, showing a cyclical pattern during the seminiferous epithelial cycle. Thus, the extracellular concentration of GDNF in a given area of the seminiferous tubule may vary with time [10]. Intriguingly, GDNF has also been demonstrated to stimulate chemotaxis in both normal, transformed cells [11]–[14] and seminoma cells [15]. Here, we tested the hypotheses that GDNF is a chemoattractant for undifferentiated spermatogonia, including stem/progenitor cells, and that the GDNF pathway may affect proteins involved in actin cytoskeleton rearrangement in target cells.

## Materials and Methods

### Mice

All procedures were approved by the Italian Ministry for Health and conducted according to the US National Institutes of Health guidelines. C57BL/6 mice (Charles River, Italy) were used in all experiments, unless otherwise specified. For germ cell transplantation experiments, B6,129-TgR(Rosa26)26Sor, which ubiquitously express Escherichia coli lacZ, were used as donor animals, and busulfan-treated C57BL/6 mice were used as recipient animals. C57BL/6-Tg(ACTbEGFP)1Osb/J mice (Jackson Labs, England) were out-crossed with CD1 mice (Charles River, Italy) nine times to obtain CD1-Tg(ACTbEGFP)1Osb/J mice, which will be hereafter called CD1-EGFP mice. CD1-EGFP mice were crossed to DBA/2J mice (Charles River, Italy) to obtain F1 hybrid mice for the derivation of germline stem cell cultures. The animals were housed at the Histology Unit, an accredited animal facility. The animals were kept in individual cages in an environmentally controlled room (23°C, 12-h light-dark cycle) and provided with food and water *ad libitum.* All procedures were approved by the Italian Ministry for Health and conducted according to the US National Institutes of Health guidelines.

### Antibodies

The rabbit anti-VASP antibody was kindly provided by Dr. W. Witke. The rabbit anti-human GFRA1 (clone H-70) was from Santa Crutz, Italy. The goat anti-rat GFRA1 and the goat anti-mouse RET were from Neuromics, Germany. The mouse anti-human PLZF (clone 2A9) was from Calbiochem, Italy. The rabbit anti-DDX4/MVH (VASA) was from Abcam, Italy. The rat anti-mouse Thy-1 PE-conjugated (clone G7) was from BD Pharmigen, Italy. The mouse anti-α-tubulin was from Sigma-Aldrich, Italy. The goat anti-human/rat GDNF neutralizing antibody was from R&D System, Italy. The Cy3 anti-rat, the Cy3 anti-goat, the FITC anti-mouse and the FITC anti-rabbit secondary antibodies were all generated in donkeys and purchased from Jackson Laboratories, UK.

### Immunofluorescence analysis

Whole-mount immunofluorescence analysis of adult seminiferous tubules was performed as previously described [16]. Fixed seminiferous tubules were incubated with primary antibodies (anti-GFRA1, anti-PLZF and anti-VASP, dilution 1∶100) at 4°C for 16 h under constant shaking. After washing, the tubules were incubated with the species-specific conjugated secondary antibodies for two hours at room temperature. Nuclei were stained with TOTO-3 (0.2 mg/ml, Molecular Probes). GC-1 parental cells and GFRA1-transduced GC-1 cells were seeded onto an eight-well chamber slide (Lab Tek II, Nunc). Twenty-four hours post-seeding, the cells were fixed in 4% paraformaldehyde at 4°C for 10 min. The slides were pre-incubated in PBS containing 5% preimmune donkey serum, 1% BSA, and 0.1% Triton X-100 for 1 hour at room temperature. Immunostaining was performed using goat anti-GFRA1 (1∶100) antibody at room temperature for 1 hour. After washing, the slides were incubated with donkey anti-goat Cy3-conjugated antibody at room temperature for 1 hour. Nuclei were stained with TOTO-3 (0.2 mg/ml, Molecular Probes). Samples were observed using an Axioskop 2 plus microscope (Zeiss) or by confocal microscopy with a Leica TCS SP2 (Leica, Milano, Italy).

### Isolation of undifferentiated spermatogonia

Isolation of undifferentiated spermatogonia was performed as previously described [17]. The testes from two to three-month-old mice were enzymatically digested, and Thy-1 expressing cells were selected using a Magnetic Activated Cell Sorter (MACS, Miltenyi Biotech). Single-cell suspensions were incubated with PE-conjugated anti-Thy-1 antibody and, after washing, with anti-PE microbeads (Miltenyi Biotech). Cells were separated on a column placed in the magnetic field of a MACS separator. Unselected cells, Thy-1-positive and Thy-1-negative cell fractions were collected, centrifuged at 1000 rpm for 5 minutes, resuspended in DMEM and counted. The different cell fractions were employed for germ-cell transplantation assays and for semi-quantitative real-time PCR. Thy-1-positive cells were used for migration assays.

### Generation and maintenance of germline stem (GS) cell line

A GS cell line was derived from 7-day old CD1-EGFP X DBA/2J hybrid mice accordingly to a published method [7]. Cells were cultured in StemPro-34 SFM (Invitrogen) supplemented with StemPro supplement (Invitrogen), 25 µg/ml insulin, 100 µg/ml transferrin, 60 µM putrescine, 30 nM sodium selenite, 6 mg/ml D-(1)-glucose, 30 µg/ml pyruvic acid, 1 µl/ml DL-lactic acid, 10^−4^ M ascorbic acid, 10 µg/ml d-biotin, 30 ng/ml β-estradiol, 60 ng/ml progesterone (all from Sigma), 5 mg/ml bovine albumin (Biomedicals), 2 mM L-glutamine, 5×10^−5^ M 2-β-mercaptoethanol, minimal essential medium (MEM) vitamin solution, MEM nonessential amino acid solution (all from Invitrogen), 20 ng/ml mouse epidermal growth factor (Becton Dickinson, Bedford, MA), 10 ng/ml human basic fibroblast growth factor (Becton Dickinson), 10^−3^ U/ml ESGRO (murine LIF, Chemicon), 10 ng/ml recombinant rat GDNF (R&D Systems) and 1% heat-inactivated fetal calf serum (Sigma). Cultures were maintained at 37°C in an atmosphere of 7.5% carbon dioxide. Cells were routinely cultured on mitomycin C-inactivated mouse embryonic fibroblasts (MEF) and split by dissociating with 0.05% trypsin (HyClone) every 5–7 days to fresh MEF at a 1∶2 split ratio. To obtain feeder-free GS cells, the cultures were sub-cultured on laminin-treated 12-well plates for at least two weeks and split every 5–7 days [18].

### GC-1 cell line transduction

GC-1 cell lines were obtained from ATCC and cultured in DMEM (high glucose, Life Technologies), supplemented with 10% heat-inactivated fetal bovine serum (Sigma-Aldrich), 100 U/ml penicillin, 100 µg/ml streptomycin, 1× nonessential amino acids, and 1 mM Na-pyruvate (all from Life Technologies). Cells were transduced as previously described [19] using a third generation lentivirus vector (Invitrogen, ViraPower Lentiviral Expression System) that encoded a GFRA1-EGFP fusion protein. To perform the Boyden chamber assay, parental and transduced cells were serum-starved for 18 hours.

### Chemotaxis assay

The cells were assayed for their ability to migrate through a polycarbonate filter (pore size, 8 µm; Whatman International) using Boyden chambers (NeuroProbe) as previously described [15]. The cells (5×10^4^/well) were added to the upper chamber. GDNF, anti-GDNF antibody (R&D Systems), isotype control antibody or MEM alone were added alone or in combination in the lower chamber. The chambers were incubated for 5 hours at 37°C in a humidified atmosphere with 5% carbon dioxide. At the end of that time, the filters were fixed and stained. The cells from the upper side of the filter were carefully removed using a cotton swab. The cells that had migrated to the lower side of the filters were quantified by bright-field microscopy using a 40× objective, and the average number of cells per field was calculated. Mean values with SEM were used for comparison. Data are expressed as a migration index and were calculated as the fold increase over the control with no GDNF. Experiments were performed in triplicate and were repeated three times.

### Germ cell transplantation and analysis of recipient testes

Unselected and Thy-1-positive cells were isolated from B6,129-TgR(Rosa26)26Sor, kept on ice and immediately transplanted into busulfan-treated C57BL/6 mice testes. Germ cell transplantation and analyses of recipient testes were performed as previously described [17]. Recipient testes were collected 2 months after transplantation, and the number of colonies was normalized to 10^5^ injected cells. Mean values with SEM were used for comparison.

### Isolation and short-term culture of seminiferous tubules

Adult mice were killed, and the testes were removed and placed in a Petri dish containing PBS kept on ice. The testes were decapsulated and transferred to a new Petri dish. Using forceps, the tubules were gently pulled apart, carefully avoiding damaging them by squeezing or shaking. Seminiferous tubules were plated on 12-well-plates with D-MEM supplemented with antibiotics, L-glutammin, non-essential amino acid, Hepes pH 7.7, gentamicin and treated with GDNF (50 or 100 ng/ml) or left untreated for 8 hours at 37°C. Seminiferous tubules were used for quantitative real-time PCR and western blot experiments.

### Semi-Quantitative Real-Time PCR

Total RNA from MACS-selected cell fractions, from GS cells and from seminiferous tubules was employed for Real-Time PCR as previously described [10]. Briefly, total RNA was purified using TRIzol reagent (Invitrogen) and quantified. One microgram of total RNA/sample was used for cDNA synthesis with random hexamers and Transcriptor Reverse Transcriptase (Roche). In control samples, reverse transcriptase was omitted to monitor genomic DNA contamination. The cDNA was subjected to real-time analysis performed in triplicate for each sample (FluoCycleTMII SYBR Green Mix, Euroclone). Reactions were performed on Opticon2 DNA Engine (MJ Research). Primers used to detect target genes were designed and evaluated using the PRIMER3 software (http://frodo.wi.mit.edu/primer3). The amplification efficiency for each primer pair was determined by amplification of a linear standard curve of total cDNA. All primer pairs showed good amplification linearity and efficiency (>95%). Total cDNA levels were normalized against a β-actin control. Each real-time PCR assay was repeated at least two times in three different experiments. The mean values with standard error of the mean (SEM) were used for comparison. Data are presented as the fold increase compared to basal levels of expression.

### SDS-PAGE and western blot analysis

Seminiferous tubules, GS cells and GC-1 cells were homogenized in lysis buffer (50 mM Hepes pH 7.4, 2 mM EGTA, 15 mM MgCl2, 1% TritonX-100, 120 mM NaCl, 12% glycerol, 1 mM dithiothreitol and a protease inhibitor mixture). Proteins from cell extracts (50 µg) were resolved with 10% SDS-PAGE and transferred to Hybond C-extra nitrocellulose. Blots were incubated with primary antibody overnight at 4°C, and antigen–antibody complexes were revealed by enhanced chemiluminescence (ECL; Santa Cruz Biotechnology). Western blot signals were scanned, and the band intensity was quantified using Aida software, version 2 (Raytest). The densitometric signal for experimental values was divided by the signal for the loading control protein (tubulin), and this value was expressed as fold-change over the control with no GDNF.

### Statistical analysis

Statistical analysis was performed using SigmaPlot 11.0 (San Jose, CA). Data are expressed as the mean ± standard error of the mean (SEM). Statistical analysis was performed by ANOVA, and all pairwise multiple comparison procedures were performed by the Student-Newman-Keuls Method, with p values less than 0.05 considered significant.

## Results

### GDNF induces directional migration of undifferentiated spermatogonia

**Figure 1:**
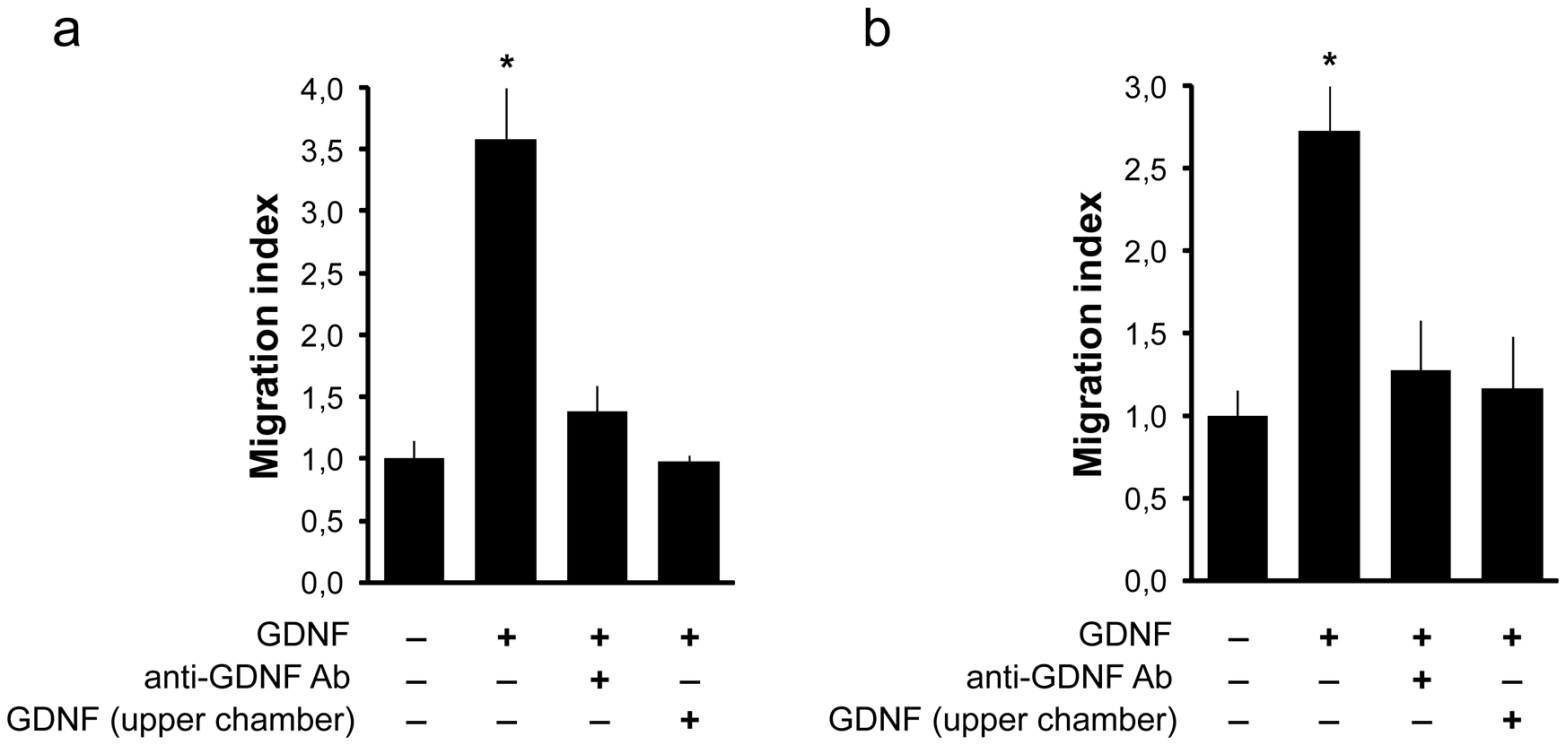
GDNF induces the migration of undifferentiated spermatogonia. Cell migration was evaluated using the Boyden chamber assay, as detailed in the Materials and Methods section. GS cells subcultured on laminin (a) or Thy-1-selected cells (b) were treated at 100 ng/ml GDNF, 15 µg/ml neutralizing antibody, or, to abrogate the GDNF gradient in the Boyden chamber, 100 ng/ml GDNF was added in both the upper and lower chambers. The results are shown as the migration index, which was calculated as the fold increase over the control with no GDNF. Data are expressed as the mean ± SEM from n = 3 experiments, measured in triplicate. *P<0.05 vs. control with no GDNF (one-way ANOVA, Student-Newman-Keuls post hoc).

As GDNF is able to induce the directional migration of normal and transformed cells [11]–[14], including seminoma cells [15], we hypothesized that undifferentiated spermatogonia migrate in response to a GDNF gradient. For this experiment, we derived spermatogonial stem cell cultures (germline stem (GS) cells) from immature CD1-EGFP mice [7]. Cells were routinely cultured on a mouse embryonic fibroblasts feeder layer; however, for the migration experiments, cells were cultured for at least two weeks on laminin-treated plates to enrich undifferentiated spermatogonia (Figure S1) [18]. GS cells were employed in directional migration assays using a Boyden chamber (Figure 1a). A significantly higher fraction of cells were able to migrate in the presence of 100 ng/ml GDNF in the lower chamber compared to the control with no GDNF (p<0.05). The GDNF-neutralizing antibody (but not the isotype control antibody, data not shown), as well as the addition of 100 ng/ml GDNF in the upper chamber that prevented GDNF gradient formation in the Boyden chamber, inhibited GDNF-induced migration to the level of the control cells (Figure 1a). To further test our hypothesis, we selected germ cells from adult mouse testis using an antibody against Thy-1, which is expressed by undifferentiated spermatogonia, including the GFRA1-positive spermatogonia [17]. Thy-1 is a more effective marker for spermatogonial stem cell purification [20]. Thy-1-selected cells were tested in parallel for SSC activity by spermatogonial transplantation, gene expression analysis and directional migration assay using a Boyden chamber assay (Figure 1b and S2). Gene expression analysis demonstrated that Thy-1-selected cells expressed markers for undifferentiated spermatogonia and were significantly enriched for spermatogonial stem cell activity (p<0.001) (Fig. S2). In Thy-1-selected cells, GDNF significantly induced migration compared to the control with no GDNF (p<0.05) (Figure 1b). Migration was significantly decreased by adding a GDNF-neutralizing antibody or by adding GDNF in the upper chamber (p<0.05). Together, these data show, for the first time, that in vitro GDNF can stimulate directional migration of undifferentiated spermatogonia, including stem/progenitor cells.

### GDNF-induced migration in germ cells is mediated by GFRA1

**Figure 2:**
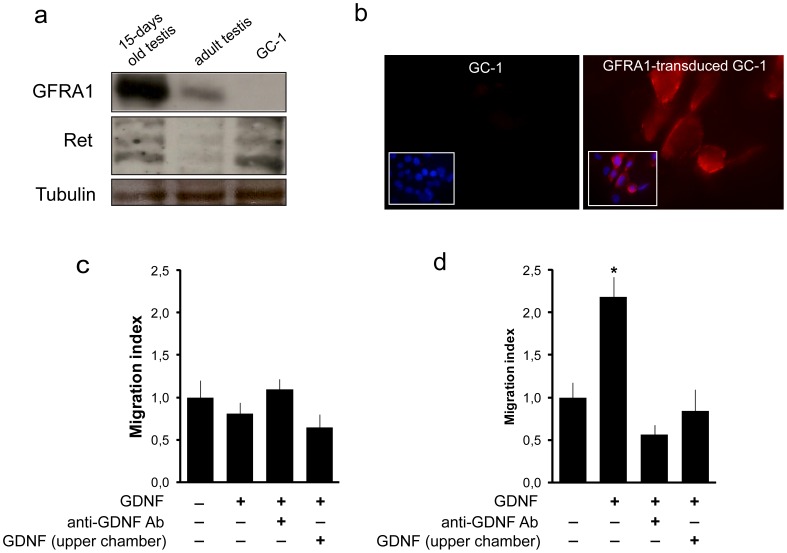
GDNF-induced migration in the GC-1 spermatogonial cell line is rescued by GFRA1 expression. (a) Western blot analysis of protein extracts from immature and adult testis and GC-1 cell lines. GC-1 cells express Ret but not GFRA1. (b) Immunofluorescence detection of GFRA1 (red) in GC-1 cells and GFRA1-transduced GC-1 cells. Nuclei were stained with TOTO-3. (c, d) GDNF-induced migration in GC-1 cells is rescued by expression of GFRA1. (c) Parental GC-1 cells, n = 3; (one-way ANOVA). (d) GFRA1-transduced GC-1 cells, n = 3; *P<0.05 vs. control (one-way Anova, Student-Newman-Keuls post-hoc).

To directly test whether GDNF-induced migration is dependent on its co-receptor, GFRA1, we analyzed GDNF chemoattraction in spermatogonia-derived GC-1 cell lines, a cell line derived from immortalized type B spermatogonia that retain markers of mitotic germ cells [21]. First, we analyzed the expression of GFRA1 and Ret (the co-receptors for GDNF) in GC-1 cells and in immature and adult testis (positive controls). Western blot analysis showed that GC-1 cells express Ret but not GFRA1 (Figure 2a), but both GFRA1 and Ret immunoreactivity were detected in immature and adult testis. To induce GFRA1 expression, GC-1 cells were transduced with a lentivirus expressing GFRA1 (Figure 2b). Next, GC-1 parental cells and GFRA1-transduced GC1 cells were employed in a Boyden chamber assay. As expected, GDNF was unable to significantly induce directional migration in GC-1 parental cells (Figure 2c). However, GDNF-induced migration in GC-1 cells was rescued by overexpression of GFRA1 (p<0.05) (Figure 2d). In GFRA1-transduced cells, both the GDNF-neutralizing antibody and the addition of GDNF in the upper chamber, inhibited GDNF-induced migration to the level of the control cells with no GDNF (Figure 2d).

### The actin-binding protein vasodilator-stimulated phosphoprotein (VASP) is expressed by undifferentiated spermatogonia

Cell movement is controlled by internal and external signals, which activate intricate signal transduction cascades that result in dynamic and localized remodeling of the cytoskeleton [22]. To test the hypothesis that the GDNF pathway may affect cytoskeleton remodeling in the undifferentiated spermatogonia, we first sought to identify proteins expressed in target cells that are known to be involved in cell migration and in reorganization of the actin cytoskeleton.

**Figure 3:**
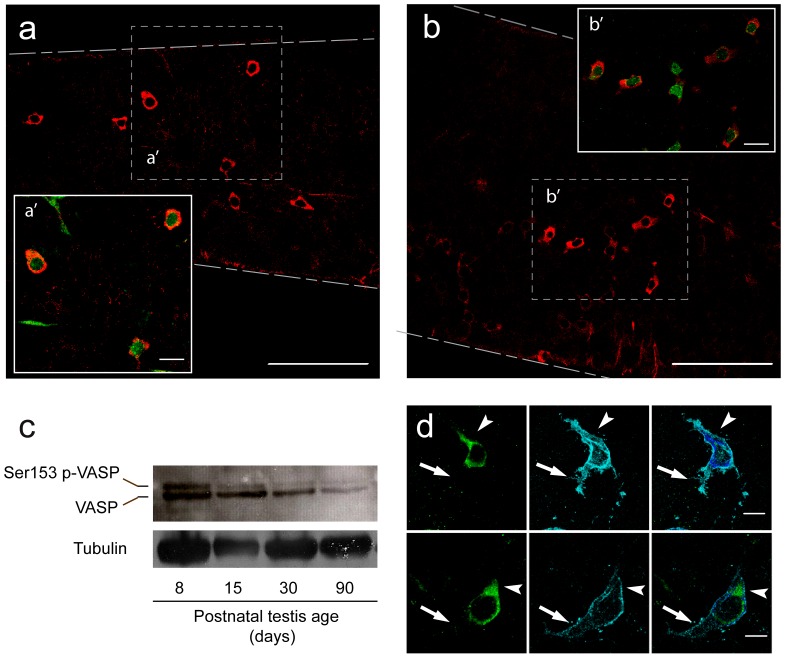
The actin-binding protein VASP is highly expressed in undifferentiated spermatogonia. (a–b) Intact seminiferous tubules were immunostained with anti-VASP (red) and anti-PLZF antibodies (green), and single Z-sections were acquired at the confocal microscope. A merging of the pictures is shown in the inset (a', b'). (c) Western blot analysis of VASP expression in extracts of testis at different ages of post-natal development. Tubulin was used as the loading control. (d) Intact seminiferous tubules were immunostained with anti-VASP (green) and anti-GFRA1 (cyan), and single Z-sections were acquired at the confocal microscope. A merging of the pictures is shown in the right column. Two polarized As cells are shown. Arrowheads indicate the VASP-positive lamellae, and arrows indicate the rear of polarized As that are negative for VASP staining. Dashed lines: outline of the seminiferous tubules. Scale bars: 100 µm in a and b; 20 µm in a' and b'; 10 µm in d.

**Figure 4:**
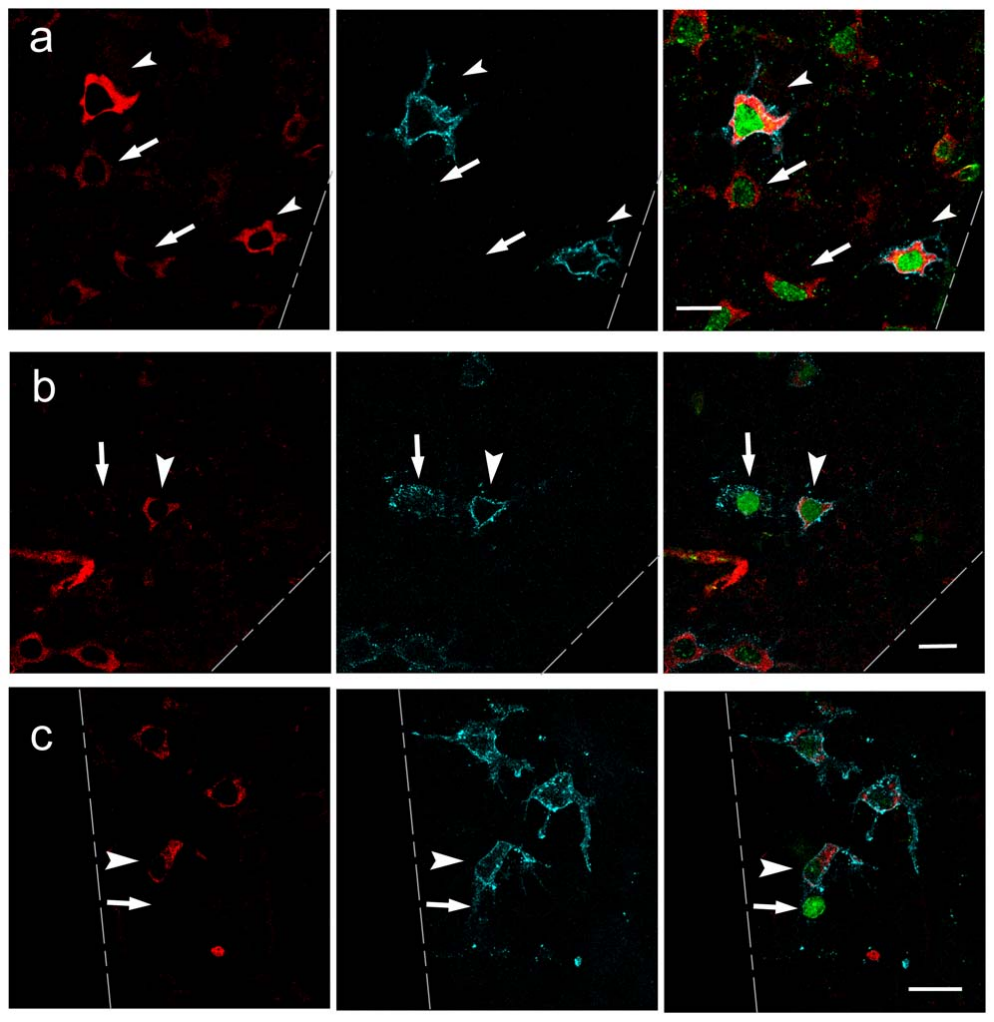
VASP correlates with GFRA1 expression in undifferentiated spermatogonia. (a–c) Intact seminiferous tubules were immunostained with anti-VASP (red), anti-GFRA1 (cyan) and anti-PLZF antibodies (green), and single Z-sections were acquired at the confocal microscope. A merging of the pictures is shown in the right column. (a) The intensity of the VASP staining in GFRA1-expressing cells (arrowheads) is higher than GFRA1 dull/negative (arrows). (b–c) Seldom we found a pair of cells (identified by PLZF expression) that were VASP and GFRA1 co-segregated in one of the two cells. Arrowhead: PLZF-positive, GFRA1-high, VASP-high cells; arrows: PLZF-positive, GFRA1-dull/negative, VASP- dull/negative. Dashed lines: outline of the seminiferous tubules. Scale bars: 20 µm.

Expression profiles of candidate proteins were analyzed by whole-mount immunofluorescence experiments on isolated tubules co-stained with anti-PLZF and anti-GFRA1. This analysis revealed that the actin-binding proteins analyzed showed a broad pattern of expression and were expressed in all germ cells types and somatic cells (data not shown). Surprisingly, the actin-binding protein VASP was highly expressed in PLZF-expressing spermatogonia (Figure 3a, b). Western blot analysis of proteins extracts from testes at different postnatal ages revealed a doublet of bands at 46–50 kDa. Murine VASP can be phosphorylated on three different residues by protein kinase A and protein kinase G [23]. Phosphorylation of Ser153 leads to a shift in the apparent molecular mass of VASP, but phosphorylation of Ser235 and Thr274 does not alter VASP mobility in SDS-PAGE [23]. As the antibody employed in our analysis detects all VASP forms, the upper band corresponds to VASP phosphorylated at Ser153 (Ser153 p-VASP), and the lower band corresponds to total VASP, in either the un-phosphorylated or Ser235/Thr274 phosphorylated forms. The VASP doublet was detected throughout testis development, but, when compared to immature testis, its expression levels decreased in adult testis (Figure 3c). Triple immunostaining with VASP, PLZF and GFRA1 antibodies revealed that, in some polarized As cells, VASP was found on large lamellar structures and was excluded from the long rear protrusions compatible with retraction fibers (Figure 3d). Interestingly, VASP expression levels positively correlated with those of GFRA1, particularly in As and Apr spermatogonia, and was down-regulated in Aal (Figure 4a). In most of the Apr, VASP and GFRA1 were co-expressed in the two cells of the doublet. We seldom found a pair of cells, as identified by PLZF expression, where VASP and GFRA1 were co-segregated in one of the two cells (Figure 4b, c).

### VASP is regulated by GDNF in undifferentiated spermatogonia

**Figure 5:**
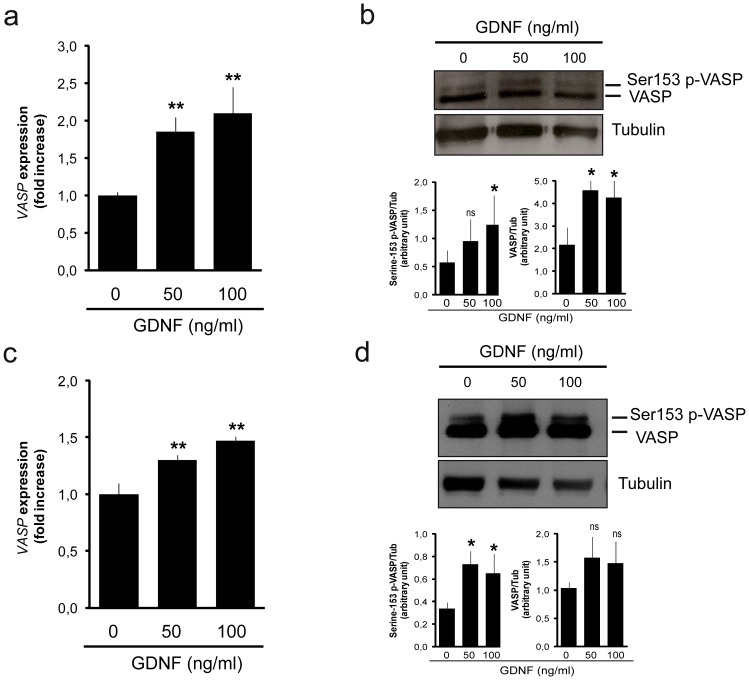
VASP is regulated by GDNF both at transcriptional and post-transcriptional levels. Intact seminiferous tubules (a–b) or GS cells (c–d) were cultured in the presence of an increasing concentration of GDNF. VASP expression level was analyzed at the mRNA level by real time PCR (a, c) and at the protein level by western blot analysis (b, d). (b, d) Representative blots are shown, and histograms at the bottom of the blots show the average data obtained by densitometric analysis of the Ser153 p-VASP (left) and the VASP (right) immunoreactive bands from three different experiments. The results (mean ± SEM) are expressed as density in arbitrary units. *P<0.05 vs. control; **P<0.001 vs. control; ns: not significant vs. control.

As GFRA1 is the co-receptor for GDNF and the expression level of VASP positively correlated with GFRA1, we hypothesized that VASP transcriptional levels are regulated by GDNF itself. To test this hypothesis, seminiferous tubules isolated from adult testis were cultured for 8 hours with increasing concentrations of GDNF and analyzed for VASP expression. Real-time PCR analysis indicated that VASP mRNA was up-regulated by GDNF in a dose-dependent fashion (Figure 5a). Western blot analysis performed on protein extracts from parallel samples showed that GDNF induced an increase in VASP levels, in both bands of the doublets (Figure 5b). We next asked whether a similar regulation also takes place in GS cells. GS cells, cultured on laminin-treated plates, were left without GDNF for 16–18 hours. Afterward, cells were treated for 8 hours with increasing concentrations of GDNF and VASP was analyzed by real-time PCR and western blot in parallel samples. Again, real-time PCR analysis and western blots showed that VASP mRNA and VASP doublets were up-regulated by GDNF in GS cell cultures (Figure 5c, d). Together, these data show that VASP is possibly regulated by GDNF at both the transcriptional and post-transcriptional level.

## Discussion

It is well established that GDNF is key factor for self-renewal and differentiation of spermatogonial stem cells, both in vivo and in vitro [24]. However, the underlying mechanisms are incompletely understood. The effect of GDNF as a chemoattractant, first recognized in epithelial cells [13], is now well documented in different cell types and cells that are both normal and transformed [11]; [12]; [14]; [15]. Here, we show that GDNF induces the directional migration of freshly selected undifferentiated spermatogonia, as well as germline stem cells in culture. In Boyden chamber migration assays, the GDNF effect was specific, and it was completely reverted by a neutralizing anti-GDNF antibody. We also show that GDNF stimulates chemotaxis and not chemokinesis (i.e., random migration). When the GDNF gradient was abrogated by adding GDNF to both the upper and lower chambers, the number of migrated cells was similar to the control without GDNF in the lower chamber. We have recently shown that the GDNF co-receptor [15] GFRA1 is over-expressed in human *in situ* carcinoma (CIS) and in intratubular and invasive seminoma [15]. Interestingly, GDNF does not affect cell survival and proliferation in the seminoma cell line, but it does induce directional migration and promotes invasive behavior. GDNF-induced seminoma cell migration appears to be mediated by the Src and MEK pathways but not by the PI3K pathway [15]. Our data indicate that the chemoattractant activity of GDNF is conserved in both normal and transformed germ cells.

The identification of chemoattractant gradients in the extracellular space and how they evolve with time is important for understanding the signals driving cellular chemotaxis in intact tissues [25]. In the mouse testis, GDNF is synthesized in Sertoli cells, but the molecular mechanisms controlling its secretion or distribution in vivo are unknown at present. There are no data concerning the putative GDNF gradient. However, it has been recently found that expression and secretion of GDNF are stage-dependent, showing a cyclical pattern over the seminiferous epithelial cycle [10]; [26]; [27]. In adult mouse testis, GDNF secretion is maximal at stages II–VI and reaches a minimum at stages IX–XI, in parallel to the stage-specific response of Sertoli cells to FSH [10]. The cyclic production of GDNF over the 8.5 days of the seminiferous epithelial cycle suggests that the extracellular concentration of GDNF in a given area of the seminiferous tubule may vary with time, setting the conditions for gradient formation [10].

GDNF interacts with heparan sulfates (HS), which are required for GDNF signaling through the GFRA1–RET complex [28]–[30]. More recently, it has been shown that syndecan-3, a transmembrane heparan sulfate proteoglycan, is a novel receptor for GDNF, as well as for neurturin and artemin, which are two other members of the GDNF family of ligands [31]. Binding of GDNF to syndecan-3 requires its HS chains and triggers the activation of Src family kinases, leading to cell adhesion and spreading. Syndecan-3 also mediates GDNF-dependent migration and differentiation of cortical GABAergic neurons. Based on this experimental observation, it has been proposed that GDNF may act as a diffusible free protein that activates the canonical GFRA1-RET co-receptors pathway. Alternatively, GDNF may be immobilized on the extracellular matrix, activating the signaling through a GFRA1-independent and syndecan-3-dependent fashion [31]. In the present study, we found that the GC-1 spermatogonial cell line that lacks GFRA1 is unresponsive to GDNF. GDNF-induced migration in GC-1 cells was rescued by GFRA1 expression, suggesting that GDNF-induced migration in germ cells relies on the canonical GFRA1-RET co-receptor pathway.

In the present study, we found that PLZF-positive spermatogonia highly expressed VASP. VASP is a member of the ENA/VASP family of proteins, which are implicated in the regulation of the actin cytoskeleton and in many other cellular processes, such as cell adhesion, neuronal and fibroblast migration [32]. VASP localizes to regions of dynamic actin reorganization, such as at the leading edge of lamellipodia, at the tips of filopodia, stress fibers, focal adhesions, and at cell-cell contacts, where it has been proposed to promote actin polymerization [32]–[35]. Whole-mount immunofluorescence revealed that the VASP antibody intensely stained the cytoplasm in PLZF-expressing spermatogonia, including large lamellipodia-like structures. Moreover, we found that As and Apr spermatogonia showed higher VASP expression levels compared to Aal spermatogonia. The VASP expression of Aal spermatogonia progressively decreased to almost undetectable levels in differentiated spermatogonia. Consistent with these observations, western blot analysis performed on protein extracts of testis at different postnatal ages revealed that VASP levels were higher in immature testes compared to adult testes due to an increase in more differentiated germ cells that do not express VASP. Interestingly, VASP expression levels among undifferentiated spermatogonia directly correlated with the levels of GFRA1. The direct correlation of VASP and GFRA1 expression levels led us to hypothesize direct regulation of VASP by GDNF. We found that in vitro VASP is regulated by GDNF in seminiferous tubules and GS cells at both the transcriptional and post transcriptional levels, suggesting a similar regulation of VASP in target cells by GDNF in vivo. It has been demonstrated that VASP directly interacts with the chemokine receptor, CXCR2, which is implicated in the recruitment of leucocytes during inflammation [36]. VASP knockdown impairs CXCR2-mediated cell migration, indicating the involvement of VASP in translating the extracellular chemokine gradient into intracellular polarization cues [36]. Our data suggest that a possible mechanistic link between GDNF and VASP may be operating in GDNF-responsive cells. VASP could be involved in the actin assembly necessary to organize lamellipodial structures in target cells, in cell-cell adhesion, or in cell-substrate interactions.

The distribution of undifferentiated spermatogonia in relation to the interstitium and to other tubules is cyclic and stage-dependent. From stage III to VI, undifferentiated spermatogonia are randomly distributed over the basal lamina, but their distribution is not random in the other stages where they are primarily found in regions located closer to the interstitial tissue. It has been proposed that part of the As, Apr, and Aal clones must be moving within the seminiferous tubule to allow their cyclic distribution (random location followed by nonrandom location) [3]. More recently, these data were supported by a time-lapse analysis of GFP-labeled undifferentiated spermatogonia that were tracked in vivo for several days and were frequently found migrating over the basal lamina [4]; [5]. The ability of spermatogonia to migrate over the basement membrane is maintained in repopulating spermatogonia. Repopulating spermatogonia may be either resident germ cells surviving inflicted germ cell depletion or donor-derived spermatogonia transplanted in a recipient testis. In both cases, the recolonization process proceeds at a pace of approximately 50 microns/day or more [37]; [38]. Taking into consideration the slow doubling time of undifferentiated spermatogonia [1], the observed speed of the recolonization may imply an active migration of the repopulating spermatogonia rather than a simple reallocation of daughter cells at the border of the growing colony.

Even though the migration of undifferentiated spermatogonia is very intriguing, its physiological purpose at steady state has not been experimentally addressed. The migration of undifferentiated spermatogonia could ensure the even distribution of germ cell progeny over the basal compartment of the seminiferous tubules [3], or it may be essential for maintaining stem or progenitor cells in the right environment to ensure the self-renewal of SSCs [6]. Recent studies addressing the behavior of the spermatogonial stem cell compartment have suggested that SSCs frequently and stochastically lose their self-renewal capacity and are replaced from neighboring SSCs. In this scenario, SSCs need to migrate to repopulate the empty niches in the seminiferous tubule [4]. As GDNF is a niche-derived factor [24], our data may suggest an involvement of GDNF in either the homing or the maintenance of stem/progenitor cells in their niche.

## Supporting Information

Figure S1 **Characterization of GS cells.** GS cultures were established from 7-day old CD1-EGFP x DBA/2J hybrid mice [7] and cultured in Stem Pro medium that was supplemented with 1% FCS, GDNF, bFGF, EGF and LIF. (a) GS cells that were maintained on MEFs feeder layers formed clump-like colonies. An enlargement of a cluster of GS cells is shown in the inset. (b) GS cells sub-cultured on laminin-treated plates adhered to the bottom of the plate, forming a cluster of chain-like cells. An enlargement of GS cells is shown in the inset. (c–d) Immunophenotype analysis of GS cells cultured on laminin-treated chamber-slide. (c) VASA staining (red) reveals that all cells stain positive. The enlargement shows localization of the staining in the cytoplasm of GS cells (c'). (d) GS cells expresses GFRA1 (red). The enlargement shows that the staining decorates the cell membrane of GS cells (d'). Nuclei are counterstained with Hoechst.
(TIF)

Figure S2 **Characterization of MACS-selected Thy-1-positive cells.** Germ cells were enzymatically isolated from adult testes and labeled with anti-Thy-1 antibody, and the cell fractions were obtained by MACS selection as previously described [17]. Aliquots of unselected cells were used as controls. (a) Thy-1-positive cells were spun on a slide immunostained for PLZF (red), a marker of undifferentiated spermatogonia. Nuclei were stained with Hoechst. (b) Left: representative pictures of testis transplanted with unselected or Thy-1-positive cells at two months from transplantation; right: the histogram shows number of donor-derived colonies generated by transplantation of unselected or Thy-1-positive cells (n = 3), *p<0.001 (b) Gene expression analysis by semi-quantitative RT-PCR. Reactions were performed in parallel for each gene. The amount of specific cDNA was normalized to β-actin levels. The data (n = 3) are presented as the fold increase versus control (unselected cells), * p<0.001. Thy-1-selected cells are significantly enriched in GFRA1 expressing cells, as well as for other SSC markers.
(TIF)
