# Supplementary figures and images for: The Niche-Derived Glial Cell Line-Derived Neurotrophic Factor (GDNF) Induces Migration of Mouse Spermatogonial Stem/Progenitor Cells
Source: PLoS One. 2013 Apr 22;8(4):e59431. doi: 10.1371/journal.pone.0059431 (PMC3632550; doi:10.1371/journal.pone.0059431)

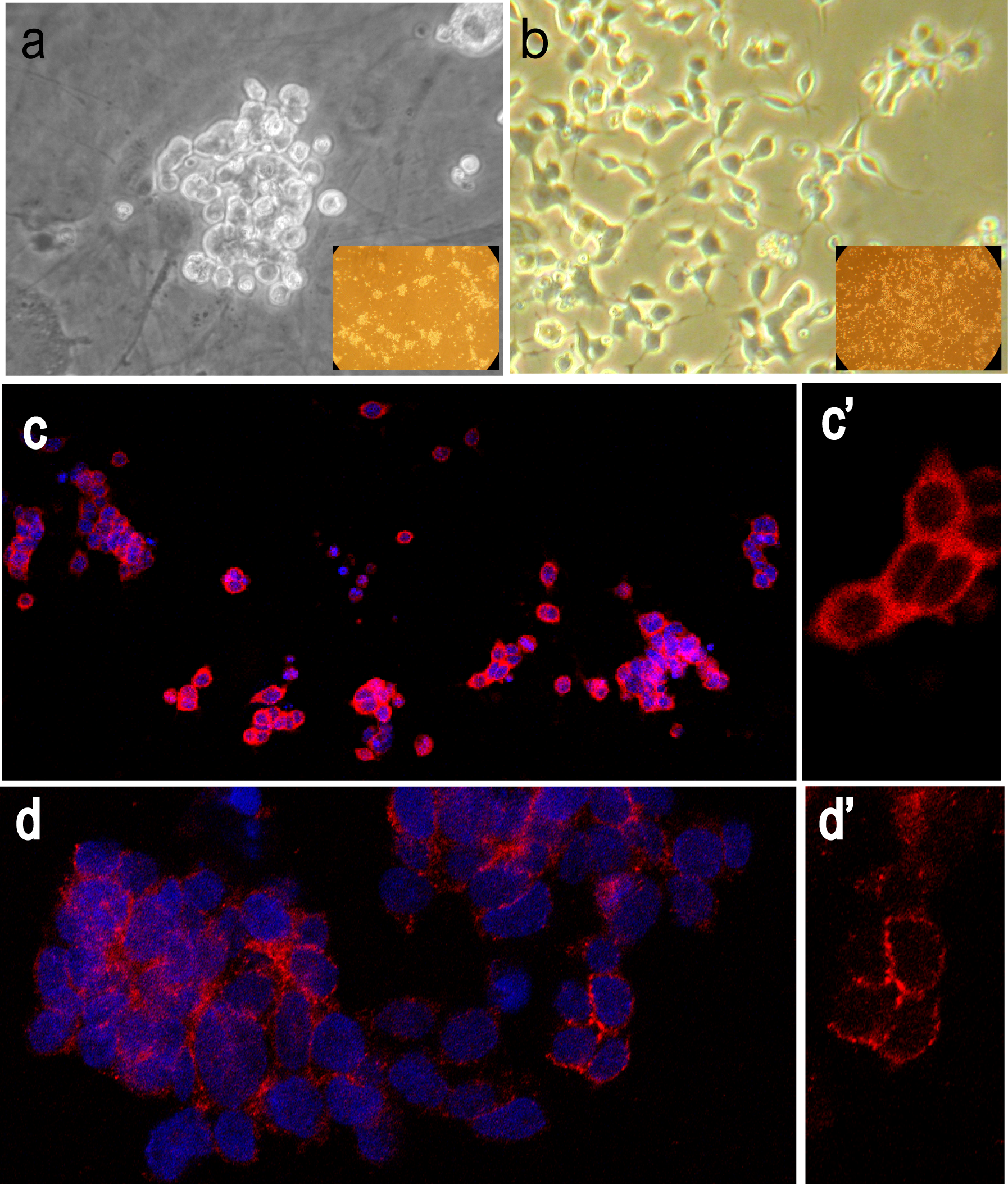

Supplement: Figure S1 — Characterization of GS cells. GS cultures were established from 7-day old CD1-EGFP x DBA/2J hybrid mice [7] and cultured in Stem Pro medium that was supplemented with 1% FCS, GDNF, bFGF, EGF and LIF. (a) GS cells that were maintained on MEFs feeder layers formed clump-like colonies. An enlargement of a cluster of GS cells is shown in the inset. (b) GS cells sub-cultured on laminin-treated plates adhered to the bottom of the plate, forming a cluster of chain-like cells. An enlargement of GS cells is shown in the inset. (c–d) Immunophenotype analysis of GS cells cultured on laminin-treated chamber-slide. (c) VASA staining (red) reveals that all cells stain positive. The enlargement shows localization of the staining in the cytoplasm of GS cells (c'). (d) GS cells expresses GFRA1 (red). The enlargement shows that the staining decorates the cell membrane of GS cells (d'). Nuclei are counterstained with Hoechst. (TIF) [file pone.0059431.s001.tif]

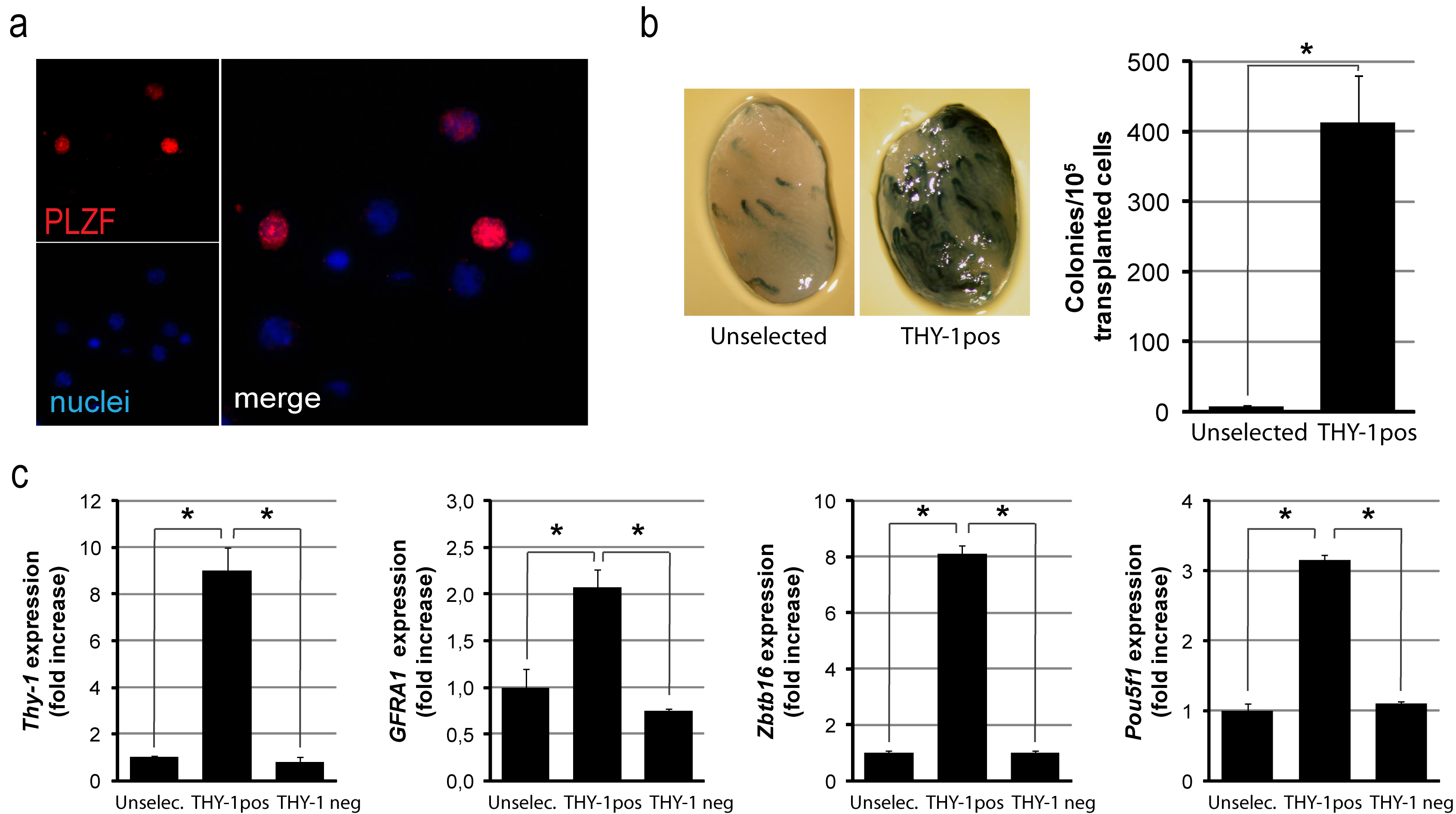

Supplement: Figure S2 — Characterization of MACS-selected Thy-1-positive cells. Germ cells were enzymatically isolated from adult testes and labeled with anti-Thy-1 antibody, and the cell fractions were obtained by MACS selection as previously described [17]. Aliquots of unselected cells were used as controls. (a) Thy-1-positive cells were spun on a slide immunostained for PLZF (red), a marker of undifferentiated spermatogonia. Nuclei were stained with Hoechst. (b) Left: representative pictures of testis transplanted with unselected or Thy-1-positive cells at two months from transplantation; right: the histogram shows number of donor-derived colonies generated by transplantation of unselected or Thy-1-positive cells (n = 3), *p<0.001 (b) Gene expression analysis by semi-quantitative RT-PCR. Reactions were performed in parallel for each gene. The amount of specific cDNA was normalized to β-actin levels. The data (n = 3) are presented as the fold increase versus control (unselected cells), * p<0.001. Thy-1-selected cells are significantly enriched in GFRA1 expressing cells, as well as for other SSC markers. (TIF) [file pone.0059431.s002.tif]
